# Expression of ^2^H, ^13^C, ^15^N-labeled NIST-Fab fragment in the methylotrophic yeast *Komagataella phaffii* for nuclear magnetic resonance studies

**DOI:** 10.1051/epjconf/202328601003

**Published:** 2023-10

**Authors:** Kinlin L. Chao, William B. O’Dell, Tsega L. Solomon, Robert G. Brinson, John P. Marino, Zvi Kelman

**Affiliations:** 1 Institute for Biosciences and Biotechnology Research; 2 National Institute of Standards and Technology and the University of Maryland; 3 Biomolecular Labeling Laboratory, Rockville, MD 20850, USA.

## Abstract

Labeling of proteins with deuterium is an essential tool in overcoming size limitations in the application of nuclear magnetic resonance (NMR) spectroscopy to proteins larger than 30 kilodaltons (kDa). A non-originator antigen-binding fragment (Fab) of NIST RM 8671 NISTmAb, so called yNIST-Fab, is a ~ 50 kDa protein, with 5 native disulfide linkages, that can be expressed in properly folded form in methylotrophic *Komagataella phaffii* (formerly *Pichia pastoris*). Further, the *K. phaffii* host can support the production of perdeuterated yNIST-Fab which is necessary to obtain well-resolved TROSY-based triple-resonance NMR spectra for chemical shift assignment of the peptide backbone resonances. Here, we examined growth conditions and effects of media composition to maximize biomass generation and expression yield of the ^2^H, ^13^C, ^15^N-enriched NIST-Fab fragment. Triple-labeled yNIST-Fab with ~93% deuteration reduced the ^1^H_N_, ^15^N and ^13^C-linewidths in the NMR spectra, allowing sequential NMR assignment of backbone resonance - a key step toward sequence-specific structural and dynamic studies of Fab fragments and intact antibodies.

## 1 Introduction

Monoclonal antibodies (mAbs) are a vital therapeutic platform for the treatment of cancers and various diseases with 162 antibody therapies currently approved by drug regulatory agencies in the US, Europe, Japan, and China [1]. The cluster of differentiation 3 (CD3) protein complex and T cell co-receptor appears the most common target with 10 approved antibodies for organ transplant rejection (The Antibody Therapies Database, Umabs-DB, https://umabs.com). Yet the anti-CD3 antibody development pipeline continues with 6 in Phase-3, 48 in Phase-2, 87 in Phase-1, and 319 in Preclinical trials. It is critical to evaluate the stability and efficacy of these pharmaceutical proteins during manufacture, formulation, and storage. The majority of therapeutic mAbs are of the immunoglobulin G1 (IgG1) subclass. IgG1s are 150 kDa tetrameric proteins consisting of two heavy chains (HC) and two light chains (LC) covalently linked by disulfide bonds. The heavy chain is divided into variable (V_H_) and constant (CH_1_, CH_2_ and CH_3_) domains and light chains into V_L_, and C_L_ domains. Antibodies are comprised of functional antigen binding (Fab) and crystallizable (Fc) regions. The Fab portion, comprising V_H_, CH_1_, V_L_ and C_L_ domains, binds specifically to the antigenic epitope. The Fc region, disulfide linked CH_2_ and CH_3_ domains with N-linked glycosylation at asparagine residue 297, interacts with antibody receptors on the cell surface.

Fingerprinting methods using nuclear magnetic resonance (NMR) spectroscopy at natural isotopic abundance is gaining increased acceptance for assessment of the critical quality attributes (CQA) of higher order structure (HOS) for protein-based therapeutics [2–6]. Spectral changes, in the form of shifted cross peaks or the appearance and disappearance of signals, reflect HOS perturbations, can arise from misfolding, aggregation, degradation, and/or chemical modifications of proteins. NMR fingerprinting methods have been applied to many different protein modalities, including peptides, small proteins, and intact mAbs [7–10]. Despite the success of NMR fingerprinting methods for industrial applications, any observed spectral perturbations for intact mAbs cannot be directly traced to specific structural elements without the availability of spectral assignments.

NMR resonance assignment opens the possibility for detailed structural analysis including sequence specific assessment of HOS perturbations of biologics. Although there are relatively few backbone assignments for proteins over 50 kDa, the HOS of intact mAbs can be considered modular. Previous work has established that the constituent Fc and Fab domains retain the HOS of the parent molecule with only minor chemical shift perturbations observed for residues near in the hinge region [11]. Until our recent publication of NMR assignments of a Fab domain [12], only assignments of the Fc domain were available including from glycosylated and aglycosylated versions from CHO (Chinese Hamster Ovary) and *Escherichia coli* expression systems [11, 13]. The first step in chemical shift assignment is to conduct a suite of triple-resonance NMR experiments to correlate the chemical shifts of the amide proton (H_N_) with the chemical shifts of Cα and Cβ [14]. While these experiments require uniformly ^13^C and ^15^N isotope labeling, proteins larger than 30 kDa also need deuterium labeling to eliminate efficient proton relaxation pathways and allow for optimal implementation of the transverse relaxation optimized spectroscopy (TROSY) element within pulse sequences [15], leading to sensitivity enhancements for high resolution NMR spectra. Despite these technical requirements for application of NMR to Fabs, prior to this study no expression systems have been described for the production of a properly folded, uniformly labeled ^2^H, ^13^C, ^15^N Fab domain.

To address the need for a ^2^H, ^13^C, ^15^N Fab expression system, here we report the expression of uniformly, triply-labeled Fab domain in *Komagataella phaffii* (formerly *Pichia pastoris*). This Fab domain was derived from the publicly available NISTmAb Reference Material (RM 8671) and is referred to as yNIST-Fab due to its yeast production. The NISTmAb is an IgG1κ monoclonal antibody developed as a Reference Material (RM) by the National Institute of Standards and Technology (NIST) [16, 17]. The proper fold and disulfide bonds are formed in *K. phaffii* for the yNIST-Fab, which is then secreted into the culture media under the control of Alcohol oxidase 1 (AOX1) promoter [18]. Previous reports demonstrate that some degree of process optimization is necessary to maximize yields of cell biomass and isotopically enriched proteins in yeast from a simple minimal defined ^2^H_2_O-media [19–21]. We therefore further investigated *K. phaffii* culture growth conditions and purification protocols to achieve production of ~6.6 mg/L of triple-labeled yNIST-Fab with 93% deuteration. The TROSY-based spectra of this deuterated yNIST-Fab were acquired to demonstrate the suitability of the material for NMR experiments and enabled the first report of backbone assignments for a Fab domain [12].

## 2 Materials and methods

### 2.1 Media

### 2.2 Engineering of *K. phaffii* for improved production of yNIST-Fab

The original clone of *K. phaffii* X33 expressing yNIST-Fab (HC-Fab-1 clone) was kindly provided by Dr. Yves Aubin (Health Canada, Ottawa, ON, Canada) and was first reported by Brinson *et al.* [18]. The recombinant yNIST-Fab is a disulfide-linked heterodimer with 98% sequence identity to Fab fragment generated with papain cleavage of NISTmAb [18]. yNIST-Fab comprises residues Glutamine_1_ to Histidine_228_ of heavy chain and residue Aspartic acid_1_ to Cysteine_213_ of light chain. Because the dipeptidyl aminopeptidase A protease (STE13 gene product) failed to cleave the α-factor mating signal sequence of *Saccharomyces cerevisiae* at its recognition site, the heavy and light chain of yNIST-Fab contain vector-derived Glu-Ala-Glu-Ala residues on the N-termini of the heavy and light chains [18].

**Fig. 1.**
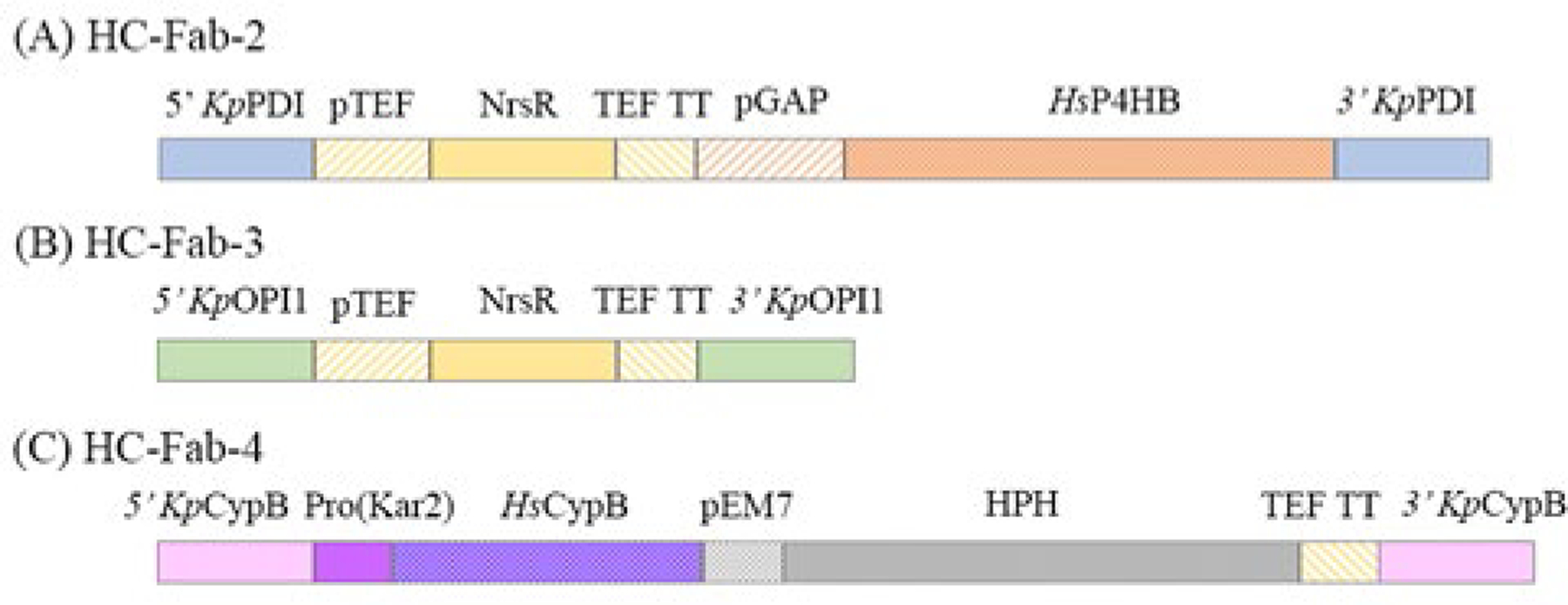
**(A**) Disruption cassette to generate HC-Fab-2 clone by replacement of *K. phaffii* protein disulfide isomerase (PDI, NCBI Gene symbol PAS_chr4_0844) gene with human prolyl 4-hydroxylase subunit β (*Hs* P4HB) to enhance disulfide bond formation of human Fab fragment. The 500 base pairs (bp) of 5’ upstream and 3’downstream flanking regions of *Kp* PDI gene are colored in blue, the NrsR gene (nourseothricin resistance, ClonNat) under the control of Translation Elongation Factor promoter (pTEF) and transcription terminator (TEF TT) in yellow, residues 17 to 508 of human (*Hs* P4HB) under *K. phaffii* glyceraldehyde-3-phosphate dehydrogenase constitutive promoter (pGAP), and the *Saccharomyces cerevisiae* α mating factor pre-peptide in orange. (**B**) Disruption cassette to generate HC-Fab-3 clone by deletion of *K. phaffii* OPI1 (PAS_chr1–1_0033) gene to enlarge endoplasmic reticulum luminal volume and to increase in antibody/fragment maturation capacity. The 500 bp of 5’ and 3’ flanking regions of *Kp* OPI1 are colored in green with NrsR gene, pTEF, and TEF TT in yellow. (**C**) Disruption cassette to generate HC-Fab-4 clone by replacement of *K. phaffii* cyclophilin B (PAS_chr1–1_0267) gene with human cyclophilin B (*Hs* CypB) to increase the rate limiting trans-cis isomerization of Proline_32_ of CH_1_ domain of Fab fragment. The 500 bp of 5’ and 3’ flanking regions of *Kp* CypB gene are colored in pink, 281 bp of 5’ promoter region of protein folding chaperone Kar2 (PAS_chr2–1_0140) in magenta, *Hs* CypB gene in purple, the HPH gene for hygromycin resistance under the pEM7 promoter in grey, and TEF TT in yellow. HC-Fab-5 was modified with both cassettes used for the construction of HC-Fab-3 and HC-Fab-4 clones.

HC-Fab-1 was modified by double-crossover homology-directed recombination gene replacement using synthetic cassettes to generate HC-Fab clones 2–5 (Fig. 1). A culture of HC-Fab-1 was made electrocompetent and transformed by electroporation according to the Thermo Fisher Scientific EasySelect *Pichia* Expression manual. Transformed colonies were selected by ClonNAT or hygromycin resistance. Genomic modifications were confirmed by gel electrophoresis and Sanger sequencing of genomic DNA PCR product amplified using primer pairs unique to specific *K. phaffii* sequences adjacent to the 5′ and 3′ homology sequences.

### 2.3 Expression of ^2^H, ^13^C, ^15^N-yNIST-Fab

All cultivations were performed in baffled shake flasks at 29 °C, 260 RPM. Cell biomass was monitored by measuring optical density at 600 nm (OD_600_). Briefly, a loopful of freshly streaked cells from a minimal dextrose agar plate was inoculated into 10 mL of 90% ^2^H_**2**_O-buffered minimal (BM) glycerol media and was grown to an OD_600_ of 5 (~ 24 h; Table 1). Approximately 0.4 mL of this culture was transferred into 100 mL of 98% ^2^H_**2**_O-BM glycerol and was incubated until OD_600_ of 7.5 (~72 h). The 98% ^2^H_**2**_O-adapted culture was expanded by inoculating ~16 mL to 500 mL of 98% ^2^H_**2**_O-BM media containing 0.5% (w/v) ^13^C_6-_glucose, and was grown to OD_600_ ~ 4.4 (~48 h). For the supplemented culture, ~17 mL of 98% ^2^H_**2**_O-adapted cells were inoculated into 500 mL of 98% ^2^H_**2**_O-BM with 0.4% (w/v) DCN-ISOGRO^®^ (herein referred to as DCN-algae extract) and 0.5% (v/v) ^2^H_8-_glycerol, and incubated until OD_600_ ~ 9–12 (~48 h). Typically, HC-Fab cultures reached saturation growth (OD_600_~20–25) in 90% ^2^H_2_O-media with 2% (v/v) glycerol, whereas the cultures in 98% ^2^H_2_O-minimal media grew slower, requiring longer incubation time to reach OD_600_ ≥ 10.

The cultures were centrifuged in sterile bottles for 20 min at 4650 x *g*, 15 °C to collect the biomass. The cell pellets were suspended in 150 mL BM media containing 0.01% (v/v) glycerol, and the centrifugation was repeated to remove traces of ^13^C_6-_glucose. The washed biomass was suspended in fresh 500 mL 98% ^2^H_**2**_O-BM media with or without DCN-algae extract supplement. The cultures were starved for ~2 h before the addition of 0.5% (v/v) methanol to induce protein expression. The cultures were fed every 24 hours with 5 mL of 50% (v/v) methanol in ^2^H_2_O (^13^C-, ^2^H_4_-, or ^13^C, ^2^H_4_-methanol). After 3 days, the culture supplemented with 0.4% (w/v) DCN-algae extract grew to slightly higher density (OD_600_ ~13.5) than the unsupplemented BM culture (OD_600_ ~11). The media was harvested by centrifugation as above.

### 2.4 Purification of secreted ^2^H, ^13^C, ^15^N-yNIST-Fab

Clarified media was adjusted to pH ~3.7 with 12 M hydrochloric acid and then applied onto a 3 mL DEAE Sephacel (Cytiva 17-0500-01) column, equilibrated with 25 mM sodium acetate, pH 4.0. The anion exchange column removed cell debris from the media and bound the majority of brown pigment from the media supplement. The flow-through from the DEAE Sephacel column was directly loaded onto a 5 mL SP Sepharose (Cytiva-17-0729-01) column, equilibrated with 25 mM sodium acetate, pH 4.0. The cation exchange column was washed with 15 mL of the same buffer, followed by 10 mL of 25 mM sodium acetate pH 4.0, 0.1 M sodium chloride. Bound yNIST-Fab, disulfide-linked light chain dimer, and monomeric light chains were eluted stepwise with 25 mM sodium acetate pH 4.0, 0.2 M sodium chloride, and then with 35 mL of 25 mM sodium acetate, pH 5.5, 0.5 M sodium chloride buffer. The separation of yNIST-Fab from the light chains shifted depending on the total protein concentration of the media. Fractions were analyzed by SDS-PAGE, and those containing yNIST-Fab were concentrated to ≥ 2 mg/mL using 10 kDa molecular weight cut-off (MWCO) centrifugal concentrators at 2100 x *g* at 12 °C. The concentrated ^2^H, ^13^C, ^15^N-protein was dialyzed in 1 L phosphate-buffered saline (PBS; 0.137 M sodium chloride, 2.7 mM potassium chloride, 10 mM dibasic sodium phosphate, 1.8 mM monobasic potassium phosphate, pH 7.3), overnight at 4 °C, prior to application onto a 5 mL IgG-CH1 affinity (Thermo Fisher Scientific 1943200) column equilibrated in PBS. The affinity column was washed with 25 mL of PBS to remove the light chain-only species, and bound yNIST-Fab was eluted with 35 mL of 0.1 M glycine, pH 3.5. These yNIST-Fab fractions were concentrated using 10 kDa MWCO concentrators to ~1 mL before applying onto a Superdex 75 10/300 GL (Cytiva 17–5174) column equilibrated with 25 mM bis-tris, pH 6.0, 0.1 M sodium chloride buffer as a polishing step. The extinction coefficient of 69,955 M^−1^ cm^−1^ (1.44 mL/mg) at 280 nm wavelength was used to determine yNIST-Fab concentration by spectrophotometry.

### 2.5 Western immunoblotting

Western blot analysis was used to monitor the time course of yNIST-Fab expression with the CaptureSelect Biotin CH1-XL conjugate antibody (Thermo Fisher Scientific 7103462100), Streptavidin-Horseradish peroxidase secondary antibody (Pierce-21130), and luminol chemiluminescent detection system. Relative signals from 2 to 20 µL of clarified media or fractions from chromatographic columns, spotted as dot blots on PVDF membranes, were quantified using BioRad Image Lab software with 10 to 150 ng of yNIST-Fab spotted on each blot as standard to normalize the signals.

### 2.6 MALDI-TOF mass spectroscopy

The intact molecular mass of yNIST-Fab was determined by the matrix-assisted laser desorption time-of-flight (MALDI-TOF) mass spectrometry with sinapinic acid (4-hydroxy-3,5-dimethyloxycinnamic acid, MilliporeSigma D-7927) as a matrix. The calculated molecular masses of unlabeled (48,436 Da) yNIST-Fab, triple-labeled yNIST-Fab (53,650 Da), and the expected masses gained from isotope incorporations were calculated from the Protein Calculator [22] (https://www.gmclore.org). The calculated molecular mass increases for 100% incorporation of ^15^N, ^13^C and ^2^H into yNIST-Fab are 561 Da, 2123 Da and 2560 Da, respectively. The intact mass for yNIST-Fab was determined to be 48,446 Da by LC-MS/MS, whereas its molecular mass by lower resolution MALDI-TOF MS was 48,784 ± 49 Da, ~348 Da higher due to peak broadening. This molecular mass of yNIST-Fab was used to estimate the isotope enrichment by MALDI-TOF mass spectrometry as follows:

(1) $$\frac{\left({MW}_{labeled}-{MW}_{unlabeled}\right)}{\left(expected\;mass\;increase\;for\;complete\;labeling\right)}\times 100=\%\;isotope\;enrichment$$

### 2.7 NMR spectroscopy

NMR spectroscopic experiments were conducted as previously described [12]. Partial refolding of ^2^H, ^13^C, ^15^N-yNIST-Fab was necessary to promote a full deuterium-to-hydrogen back exchange of backbone amides to observe the corresponding resonances [12]. For estimation of ^2^H-incorporation, the peak area from the methyl region (0.5 to 2.0 ppm) of 1D ^1^H-spectra was integrated for the unlabeled and ^2^H-labeled yNIST-Fab samples. The reference sodium 3-(tri-methyl-silyl)propane-1-sulfonate-D6 (DSS-d6, 150 µM) in each sample was used to normalize the spectra differences from data collections. All spectra were normalized to a spectrum from a 200 µM unlabeled yNIST-Fab sample with 100% protonation. ^2^H isotope enrichment was estimated by subtracting the peak area of labeled sample from corresponding peak from unlabeled yNIST-Fab with a correction for concentration difference.

## 3 Results and discussion

### 3.1 Expression of yNIST-Fab

**Fig. 2.**
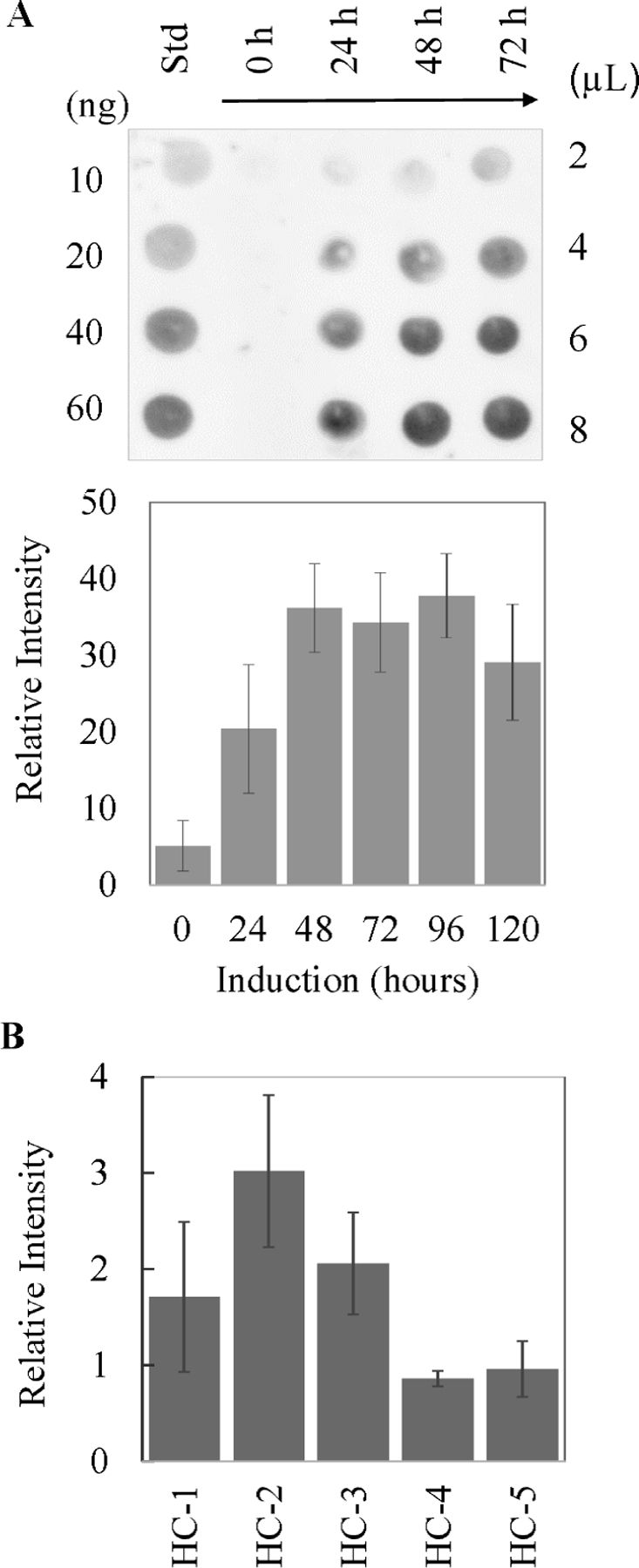
(**A**) Top panel - Western dot blot to monitor the expression of secreted yNIST-Fab as a function of induction time from HC-Fab-1 clone. Bottom panel - Quantitation of yNIST-Fab signal from 2–8 µL media on the dot blot, using the 10–150 ng yNIST-Fab standards and normalized to 1 µL media. (**B**) Secreted yNIST-Fab from the HC-Fab-1 and four modified clones (HC-Fab-2 to HC-Fab-5 as described in Materials and methods): 72 hours post-induction in H2O-miminal methanol media. The error bars represent standard deviations from three experiments.

Temperature, pH, and carbon source are parameters for optimal growth and yield of secreted recombinant protein that vary among different *K. phaffii* strains in defined minimal media [19, 20, 23, 24]. Experiments to determine the optimal growth conditions for yNIST-Fab expression were conducted in H_2_O-minimal media with methanol as the carbon source during the induction phase. The amount of yNIST-Fab present in the culture media was determined by western dot blot analysis using a CH_1_-domain primary antibody that specifically recognizes Fab. Samples were collected every 24 hours after induction (Fig. 2A, top). The time course of expression from HC-Fab-1 clone shows that a detectable amount of yNIST-Fab appeared in the media after 24 hours post-induction with slow accumulation up to 72 hours (Fig. 2A, bottom). Extending the expression from 72 hours to 120 hours did not significantly increase the secreted yNIST-Fab levels. Unlike A_2_AR GPCR [20] or *Viteroscilla* hemoglobin [25], the expression of yNIST-Fab at 23 °C during the induction phase did not change the protein yield relative to cultures grown at 29 °C (data not shown).

The HC-Fab-1 clone was engineered to improve the production of secreted yNIST-Fab by modulating the levels of endoplasmic reticulum (ER) proteins involved in protein folding and secretion [26]. Additional HC-Fab-2 to HC-Fab-5 clones were constructed to test co-expression of chaperones and modulation of the ER (Fig. 1). Of these, the HC-Fab-2 clone with constitutively overexpressed human prolyl 4-hydroxylase subunit β (P4HB), a protein disulfide isomerase, had consistently ~1.5 to 2-fold higher yield of yNIST-Fab than the original HC-Fab-1 clone (Fig. 1A & 2B). Other modifications of HC-Fab-1 clone provided limited benefit or were even detrimental to the yield of secreted yNIST-Fab (Fig. 1B–1C, 2B). An HC-Fab-3 clone was constructed to test the anticipated effect of ER expansion by deleting *K. phaffii* OPI1 gene, a known regulator of phospholipid synthesis in *S. cerevisiae* [26–28]. HC-Fab-4 clone was further constructed to examine the effect of humanizing *K. phaffii* cyclophilin B by replacement with human cyclophilin B under the control of an ER stress-inducible promoter (Fig. 1C) [29]. In addition, an HC-Fab-5 clone was constructed, which combined the engineering of HC-Fab-3 and HC-Fab-4 (Fig. 1B–1C). It should be noted that neither overexpression of *Hs* P4HB protein in HC-Fab-2 nor overexpression of *Hs* CypB protein in HC-Fab-4 and HC-Fab-5 were confirmed. It was also beyond the scope of this work to assess ER expansion in HC-Fab-3 and HC-Fab-5 clones.

**Fig. 3.**
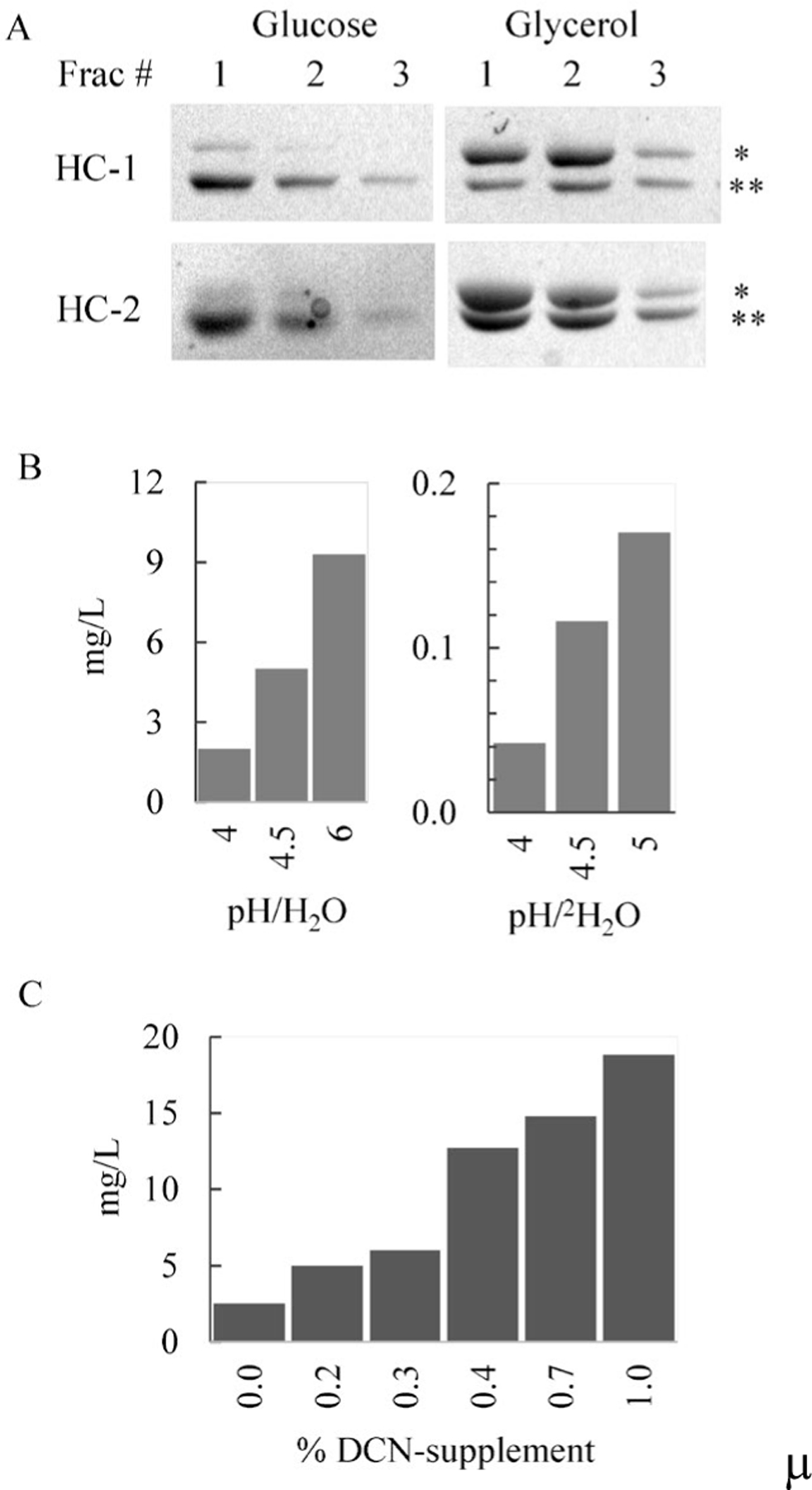
(**A**) Expression of yNIST-Fab is higher in 1% (v/v) glycerol media than in 1% (w/v) glucose as the carbon source in ^15^N, ^2^H2O-cultures. 12% SDS-PAGE under non-reducing condition of fractions from 1 mL SP sepharose column eluted with 25 mM sodium acetate, pH 4.0, 0.2M sodium chloride. HC-Fab-1 and HC-Fab-2 clones labeled as HC-1 and HC-2 with 48.8 kDa Fab and 46.2 kDa Light chain-dimer as *and **, respectively. (**B**) Yield of yNIST-Fab from H2O-media at pH 4–6 (left) and ^2^H, ^15^N-yNIST-Fab from ^15^N, ^2^H2O-media at pH 4–5 (right). (**C**) Yield of ^2^H, ^13^C, ^15^N-yNIST-Fab in ^15^N, ^2^H2O-media supplemented with (w/v) DCN-algae extract.

Glucose and glycerol are typically used as the carbon sources for the generation of yeast biomass [30, 31]. The majority of isotopic enrichment in overexpressed proteins is reported to come from the yeast biomass. A small percentage of isotope enrichment also arises from methanol during the induction phase [20, 21, 23, 24, 32]. Since yeast growth is limited in defined minimal media with methanol as the sole carbon source, a preferred procedure is to induce protein overexpression at high cell density [20]. For yNIST-Fab production, HC-Fab-1 and HC-Fab-2 cultures were cultivated in 98% ^2^H_2_O-minimal media with either glucose or glycerol to determine a preferred carbon source for biomass accumulation. After 24 h in ^15^N, 90% ^2^H_2_O-media, both HC-Fab-1 and HC-Fab-2 cultures grew to an OD_600_ of 9 with glucose, while those in glycerol media grew to a slightly higher OD_600_ of 14. This trend continued in the next adaptation step to ^15^N, 98% ^2^H_2_O-media. From the initial OD_600_ of ~0.03, the HC-Fab-1 and HC-Fab-2 cultures in glucose media grew to OD_600_ of 4.4 after 2 days, whereas the cultures in ^15^N, 98% ^2^H_2_O-media with glycerol grew to OD_600_ of 5.5 and 7.4, respectively. These data demonstrate a preference for glycerol by these yeast clones during biomass accumulation. The cell biomasses were washed to remove traces of glucose before resuspension in fresh media, and protein expression was induced with methanol for 3 days. The secreted proteins in clarified media were concentrated on an SP sepharose column, and all bound yNIST-Fab and light chain species were eluted with 25 mM acetate, pH 4.0, 0.2 M sodium chloride buffer. SDS-PAGE analysis of fractions showed the light chain dimer as the predominant product from biomass growth on glucose (Fig. 3A, Glucose). By contrast, the amount of yNIST-Fab is equal to or greater than that of the light chain species from the glycerol-derived biomasses of HC-Fab-1 and HC-Fab-2 clones (Fig. 3A, Glycerol). It is unknown why the yNIST-Fab expression is lower from the biomasses generated from glucose-media.

*K. phaffii* cultures can tolerate a pH range from 3 to 7 with an optimal growth rate at pH 6 [19]. Without continuous buffering, the media pH can drop from neutral to acidic range upon release of acetic acid if yeast cultures continue to grow during protein expression phase. For secreted expression of single chain Fv (scFv) antibody fragments in *K. phaffii,* an optimal yield of A33scFv antibody fragment was found to be at pH 3 to 4, whereas Anti-Serpin-scFv production was best at pH ≥ 6 [19, 33]. In addition, the effect of pH on yNIST-Fab expression for cultures grown in H_2_O- or ^2^H_2_O-media was examined. After 24 hours expression, the higher expression of yNIST-Fab corresponded with increasing pH for both H_2_O- or ^2^H_2_O-media types (Fig. 3B).

This result also demonstrates very low yield of ^2^H, ^15^N-yNIST-Fab from ^15^N, 98% ^2^H_2_O-media (< 0.5 mg/L from pH 6 media). This effect is expected since the slower *K. phaffii* growth rate in high [^2^H_2_O]-minimal media leads to reduction in the accumulated biomass for the protein expression on methanol [21, 34]. For *K. phaffii* expressing *Plasmodium falciparum* merozoite surface protein-1, doubling time increased from 2.5 hours in H_2_O media to 8 to 10 hours in 95% to 99.8% ^2^H_2_O-media [21]. The subsequent yield of U-^2^H, ^15^N-labeled small protein was approximately fourfold lower than that of unlabelled version [21]. Massou *et al*. reported that 140 g/L of *K. phaffii* biomass was obtained from 0 to 75% ^2^H_2_O-media, but the amount of biomass dropped to 16 g/L in 97.5% ^2^H_2_O-media [34]. A similar growth pattern with *K. phaffii* HC-Fab-2 clone was observed. In H_2_O-media, the HC-Fab-2 cultures reached saturation growth (OD_600_ ~25), while cell density remained well below OD_600_ of 10 in 98% ^2^H_2_O-minimal media. The final cell densities of these cultures ranged from OD_600_ of 4 to 10 which generated lower amounts of biomass for protein production.

### 3.2 Impact of DCN-algae extract on yNIST-Fab expression

To produce fully deuterated protein for NMR experiments, the yield of ^2^H, ^13^C, ^15^N-yNIST-Fab from ^15^N, 98% ^2^H_2_O media supplemented with DCN-labeled algae extract was investigated. The labeled amino acids and peptides, comprising ~65% of algae extract by mass, were expected to lessen the metabolic burden of *K. phaffii* Fab cultures grown in ^2^H_2_O-minimal media, especially during the protein expression phase with methanol. The presence of algae supplements (0.4%, 0.7%, 1% (w/v) DCN-algae extract) increased the cell biomass slightly (OD_600_ ~ 9 to 10) over that from the minimal media (OD_600_ ~ 6). During the induction phase with methanol, the unsupplemented control culture in 98% ^2^H_2_O-minimal media did not accumulate biomass, while the algae supplement cultures in 98% ^2^H_2_O-media grew after an initial 1 to 2 day delay. The yield of ^2^H, ^13^C, ^15^N-yNIST-Fab increased from ~2 to 8 fold (2.5 mg/L to ~18.8 mg/L at 1% algae extract) in supplemented ^15^N, 98%−^2^H_2_O media (Fig. 3C). However, above 0.4% (w/v) algae extract concentration, the ^2^H, ^13^C, ^15^N-yNIST-Fab expression level only improved by < 1.5 fold in ^15^N, 98% ^2^H_2_O-media. For comparison, ~17 to 22 mg/L of ^15^N, 15% ^13^C-yNIST-Fab were obtained from H_2_O-minimal media. Therefore, it is possible that the secretion pathway in *K. phaffii* was limiting the production of secreted yNIST-Fab rather than the availability of supplemented nutrients in ^15^N, 98% ^2^H_2_O-media. In addition, ^2^H, ^13^C, ^15^N-yNIST-Fab purified from ^15^N, 98% ^2^H_2_O-media supplemented with greater than 0.5% (w/v) isotope-labeled algae extract was irreversibly associated with brown pigment from the extract that could not be removed by dialysis or preparative size exclusion chromatography.

### 3.3 Purification of ^2^H, ^13^C, ^15^N-yNIST-Fab

**Fig. 4.**
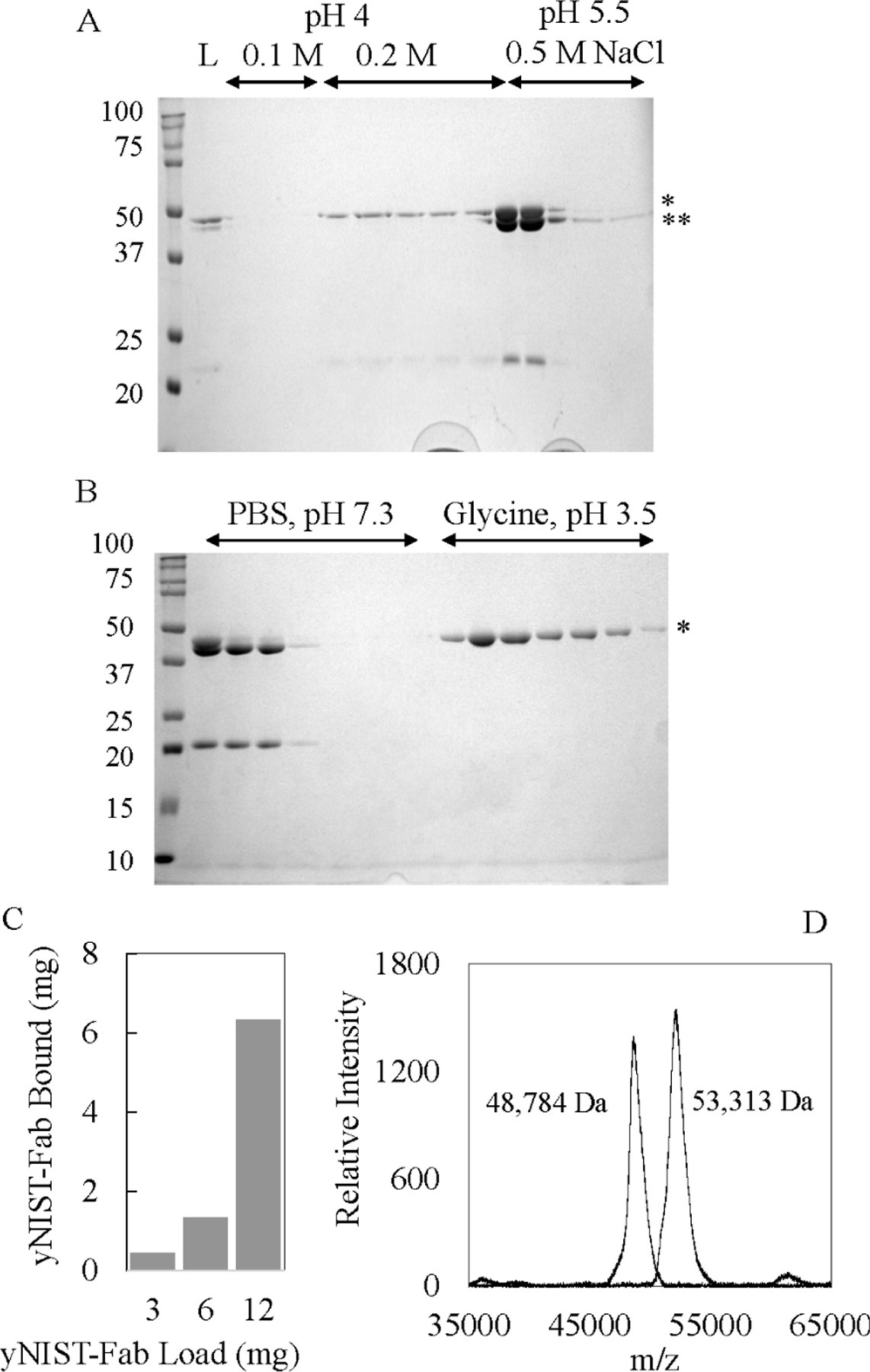
12% SDS-PAGE of ^2^H, ^13^C, ^15^N-yNIST-Fab fractions from (**A**) SP sepharose chromatography and (**B**) CaptureSelect IgG-CH1 chromatography, under non-denaturing condition with * (48.8 kDa Fab), ** (46.2 kDa Light chain-dimer) and L (load). (**C**) Binding capacity determination for 3 mL CaptureSelect IgG-CH1 column loaded with 3 mg, 6 mg, or 12 mg yNIST-Fab. Bound yNIST-Fab eluted with 0.1 M glycine, pH 3.5. (**D**) MALDI-TOF MS overlay of unlabeled (left peak) and ^2^H, ^13^C, ^15^N-yNIST-Fab (right peak).

In contrast to *E. coli* expression systems, in which many eukaryotic proteins with multiple disulfide bonds are difficult to produce, the alternative methylotrophic *K. phaffii* host offers a suitable expression system to produce full length, glycosylated antibodies, and is amendable to grow in deuterium minimal media for isotope-labeling of proteins [21, 35, 36]. However, protein expression in *K. phaffii* used twice as much ^15^N, ^2^H_2_O-medium compared against *E. coli* systems, incurring higher costs. The yeast biomass must be transferred into fresh ^15^N, ^2^H_2_O-medium prior to the induction of protein expression with methanol (see Materials and methods), highlighting the importance of capturing as much ^2^H, ^13^C, ^15^N-yNIST-Fab from the culture media and to minimize protein loss during purification. Two steps in our purification protocol affected the final yield of yNIST-Fab. Initially, pressure-driven tangential flow filters or stirred cell devices were used to concentrate liters of culture supernatant prior to purification. However, a significant amount of yNIST-Fab passed through 10 kDa MWCO regenerated cellulose or polyethersulfone (PES) membrane filters as detected by western immunoblots. As an alternative, the SP sepharose cation ion exchange resin could reliably capture all yNIST-Fab and light chain species from the supernatant while concentrating the secreted proteins greater than 100 fold. (Fig. 4A). The IgG-CH1 affinity matrix provided critical intermediate purification by removing unwanted light chain homodimer and free light chain species (Fig. 4B). Despite a highly specific association, residual yNIST-Fab was observed in the flow through of IgG-CH1 affinity column even after overnight batch binding. This resulted in multiple IgG-CH1 column experiments to capture a majority of yNIST-Fab eluted from the S Sepharose column. The amount of bound yNIST-Fab to 3 mL IgG-CHI column increased proportionally to the amount of loaded protein (Fig. 4C). This unexpectedly low capture capacity was resolved by concentrating the input material to ≥ 2 mg/mL (Fig. 4C), suggesting that protein concentration was a factor in the effective dynamic binding capacity of the IgG-CH1 affinity matrix for yNIST-Fab.

### 3.4 Estimation of stable isotope incorporation by MALDI-TOF Mass-spectroscopy

**Fig. 5.**
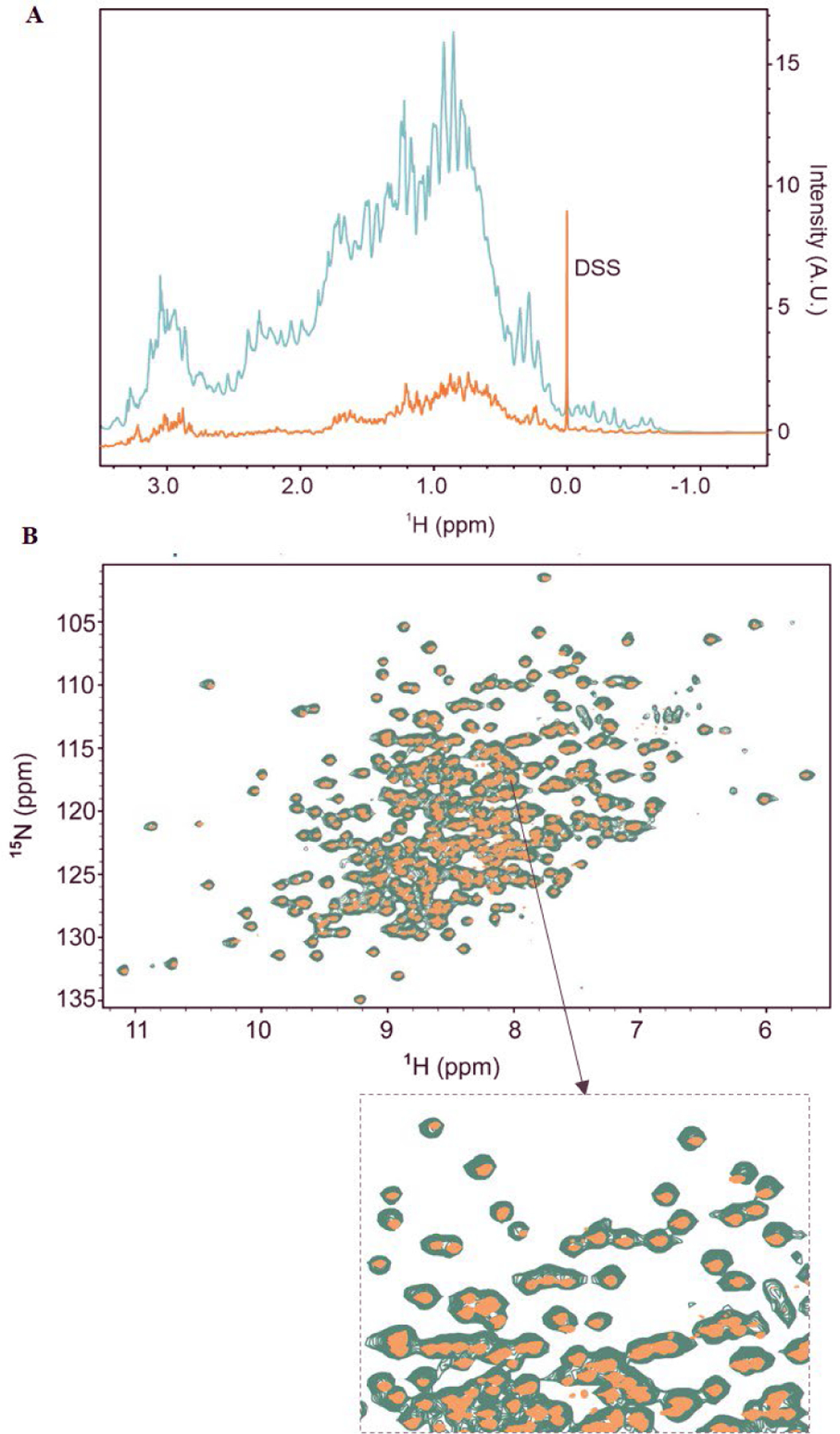
(**A**) 1D ^1^H-NMR spectra overlay of 490 µM U-^13^C, ^15^N-yNIST-Fab (cyan), 500 µM ^2^H, ^13^C, ^15^N-yNIST-Fab (gold), and DSS (sodium 3-(trimethylsilyl)propane-1-sulfonate-D6 reference) at 37 °C and 900 MHz. (**B**) 2D ^1^H-^15^N-HSQC of U-^13^C, ^15^N-yNIST-Fab (cyan) and TROSY-HSQC of ^2^H, 13C, ^15^N-yNIST-Fab (gold). Inset shows an expanded region of the spectrum from 105 to 120 ppm in the nitrogen dimension and 6.5 to 9.2 ppm in the proton dimension.

To estimate the stable isotope incorporation, the intact masses for a series of stable-isotope-labeled yNIST-Fab were measured using MALDI-TOF MS (Table 2). This procedure for stable-isotope incorporation was previously established for the EGF domain of thrombomodulin with >98% ^15^N-enrichment [24]. For ovine interferon-tau proteins, the combined ^13^C, ^15^N incorporation varied with labeling protocols: 68 to 83% for one protocol and 99% for another protocol [23]. Uniform ^15^N- and 90% ^13^C-labeling has been reported for eukaryotic seven-transmembrane rhodopsin expressed in *K. phaffii* for solid state NMR studies [30]. This approach allowed the evaluation of the contribution of ^2^H_2_O media and methanol to overall isotope enrichment of yNIST-Fab. The intact masses of unlabeled and ^2^H, ^13^C, ^15^N-yNIST-Fab from MALDI-TOF analyses are 48,784 ± 49 Da (Fig. 4D, Table 2, sample-1) and 53,313 ± 52 Da, respectively (Fig. 4D, Table 2, sample-14). The 1D ^1^H spectra of protonated ^13^C, ^15^N- and ^2^H, ^13^C, ^15^N-yNIST-Fab with ~93% deuteration show reduction in ^1^H signals (Fig. 5A). ^2^H-yNIST-Fab from 98% ^2^H_2_O-media with methanol as the carbon source has a molecular mass of 51,189 ± 125 Da (Table 2, sample-2), corresponding to an average substitution of ^2^H at 94% of non-exchangeable positions in yNIST-Fab. Deuteration of human µ-opiate receptor in *K. phaffii* showed that 80 ± 5% deuteration of amino acids came from ^2^H_2_O-media with methanol, while 20 ± 10% of deuteration of amino acids was derived from ^2^H_4_-methanol as the carbon source in H_2_O-media [34]. Similarly, the presence of ^2^H_4_-methanol in ^2^H_2_O-media contributed an additional 9% deuteration of the C-terminal fragment of the *P. falciparum* merozoite surface protein-1 from 72% to 81% enrichment in ^2^H_2_O-media with protonated methanol as carbon source [21]. The deuterated levels of ^2^H, ^15^N-labeled yNIST-Fab (Table 2, samples 3–4) from either methanol or ^2^H_4_-methanol were the same (~85%) within experimental error, suggesting minimal contribution from ^2^H_4_-methanol. Likewise, 1D ^1^H-NMR experiments of ^2^H, ^13^C, ^15^N-yNIST-Fab from ^15^N, 98% ^2^H_2_O-media with 0.2 to 0.4% (w/v) DCN-algae extract supplement and ^2^H_4_-methanol (Table 2, samples 5–7) showed ~85 to 87% deuteration with no apparent contribution from those added carbon sources. By comparison, ^2^H, ^13^C, ^15^N-yNIST-Fab from ^15^N, 98% ^2^H_2_O media with 100% ^13^C-methanol gave a deuteration level of 58% from 1D ^1^H NMR analysis (Table 2, sample 13). ^13^C, ^2^H_4_-methanol was used to produce ^2^H, ^13^C, ^15^N-yNIST-Fab (Table 2, sample 14) with as high a level of deuteration as possible from ^15^N, 98% ^2^H_2_O media, 0.4% (w/v) DCN-algae extract. The 1D ^1^H NMR analysis revealed this ^2^H, ^13^C, ^15^N-yNIST-Fab (sample-14) has 93% deuteration. With one exception, the 1D ^1^H NMR and MALDI-TOF MS analyses showed ~85% to 94% ^2^H-enrichment for most deuterated yNIST-Fab samples.

In addition to optimizing ^2^H labeling, incorporation of ^13^C was also investigated to determine the level of contribution from cell biomass and from methanol. Wood and Komvies [24] reported that 70% of ^13^C-enrichment in thrombomodulin’s EGF domain comes from cell biomass grown in ^13^C-glucose and the remaining 30% from ^13^C-methanol during the induction phase. In contrast to ^2^H-enrichment, the ^13^C-methanol and ^13^C-labeled peptides and amino acids of algae extract contribute equally to the ^13^C-incorporation into yNIST-Fab (Table 2, samples 8–13). When expressed in ^15^N, H_2_O-minimal media with 20%^13^C/80%-methanol as carbon source, mass spectrometry analysis revealed ~14% to 16% ^13^C-enrichment for ^13^C, ^15^N yNIST-Fab (Table 2, sample 12). The ^13^C-incorporation increased to ~36% in ^13^C, ^15^N-yNIST-Fab purified from cultures grown with 100% ^13^C-methanol (Table 2, sample 13). When the ^13^C-source was solely from the *K. phaffii* biomass grown in ^2^H_2_O-media supplemented with CN- or DCN-algae extract, the ^13^C-enrichments in ^2^H, ^13^C, ^15^N-yNIST-Fab (Table 2, samples 6 and 7) are estimated to be in 24% to 28% range. In the presence of both ^13^C-methanol and CN/DCN-algae extracts, the ^13^C-enrichments were greater than 50% for ^2^H, ^13^C, ^15^N-yNIST-Fab samples 8–11 (Table 2). The TROSY-based backbone NMR experiments for resonance assignment were conducted on the ~85% ^13^C-enriched ^2^H, ^13^C, ^15^N-yNIST-Fab sample-14 [12].

### 3.5 Quality of amide backbone spectra from triple-labeled yNIST-Fab

A total of 422 non-proline amide ^1^H, ^15^N cross peaks are expected from 48.4 kDa U-^15^N, 20% ^13^C-yNIST-Fab, and the corresponding backbone fingerprint is congested with overlapping cross peaks [18]. The reduction of the amide proton and nitrogen linewidths is critical in obtaining well-resolved TROSY spectra for the sequence specific assignment [14]. The overlay of 2D ^1^H,^15^N spectra of ^13^C, ^15^N-yNIST-Fab (sample 11) and ^2^H, ^13^C, ^15^N-yNIST-Fab (sample 14) illustrate a high-quality backbone fingerprint of the deuterated sample resulting from narrow linewidths with well dispersed ^1^H and ^15^N dimensions (Fig. 5B). The backbone chemical shift assignments of NIST-Fab were made using this triple-labeled yNIST-Fab sample, representing a step toward in-depth molecular assessment of the HOS of NIST-Fab fragment.

## 4 Conclusion

Here we report a protocol for optimized expression and purification of highly deuterated, ^13^C, ^15^N-enriched Fab fragment of the NISTmAb RM 8671 reference IgG1κ with native disulfide bonds in the *K. phaffii* expression system. U-^15^N, 93% ^2^H, 85% ^13^C-yNIST-Fab enabled application of TROSY-based NMR experiments for the assignment of the Fab backbone chemical shifts, opening the door to detailed, sequence-specific structural analysis of Fab fragments as well as intact mAbs. This simple protocol can be easily implemented for the isotope labeling of proteins in *K. phaffii* using commonly available lab equipment. We showed that yeast host cell engineering and the addition of algae supplement to the 98% ^2^H_2_O-minimal media contribute to obtaining a high expression of ^2^H, ^13^C, ^15^N-yNISTFab sample for NMR studies. Finally, our results demonstrate MALDI-TOF mass spectrometry as a reliable method in estimating isotope-incorporation levels..

We are indebted to Genevieve Gingras and Yves Aubin (Centre for Biologics Evaluation, Biologics and Genetic Therapies Directorate, Health Canada, Ottawa, ON, Canada) for sharing the original clone of *K. phaffii* X33 expressing yNIST-Fab.

## Figures and Tables

**Table 1.** Components for supplemented minimal growth media.

Buffered minimal (BM) Media without supplements 0.1 M Potassium phosphate, pH 6.0 0.34% (w/v) Yeast nitrogen base without amino acids and ammonium sulfate (Research Product International Y20060) 1% (w/v) 15N-Ammonium sulfate (MilliporeSigma 299286) 0.00004% (w/v) Biotin (Thermo Fisher Scientific BP-232–1) 0.0002% (v/v) Antifoam 204 (MilliporeSigma A6426) 98% (v/v) 2H2O (Cambridge Isotope Laboratories DLM-4–99.8–1000) 1% (v/v) glycerol
Supplements added to above BM media during the biomass generation and protein expression phases. 1 x Minimum Essential Medium (MEM) essential amino acids (Corning 25-030-CI) 1 x MEM nonessential amino acids (Corning 25-025-CI) 1 x MEM vitamins (Corning 25-020-CI) 1 x Pichia Trace Mineral 1 salts (VWR 241) 0.2 – 1% (w/v) ^2^H, ^13^C, ^15^N-ISOGRO^®^ (MilliporeSigma DCN-IG 608297–125)
Carbon source during protein expression 0.5% (v/v) ^13^C-methanol (MilliporeSigma 277177), ^13^C, ^2^H4-methanol (MilliporeSigma 293865), or ^2^H4-methanol (CIL DLM-24RG)

**Table 2.** Molecular mass of unlabeled and isotope-labeled yNIST-Fab by MALDI-TOF MS for the estimation of isotope-enrichment.

Sample#	Methanol	MALDI-TOF		NMR (^2^H)
		Daltons	% Isotope	
1) -	MeOH	48,784±49	-	nd
2) ^2^H	MeOH	51,189±125	94% ^2^H	nd
3) ^2^H^15^N	MeOH	51,513±47	85% ^2^H	nd
4) ^2^H^15^N	d_4_-MeOD	51,476±39	83% ^2^H	nd
5) ^2^H^15^N, 0.2% DCN^a^	d_4_-MeOD	51,555±73	1% ^13^C^a^	85%
6) ^2^H^15^N, 0.3% DCN^a^	d_4_-MeOD	52,035±44	24% ^13^C^a^	86%
7) ^2^H^15^N, 0.4% DCN^a^	d_4_-MeOD	52,110±52	28% ^13^C^a^	87%
8) ^2^H^15^N, 0.4% DCN^a^	^13^C-MeOH	52,573±123	50% ^13^C^a^	nd
9) ^2^H^15^N, 0.7% DCN^a^	^13^C-MeOH	52,585±79	50% ^13^C^a^	nd
10) ^2^H^15^N, 1.0% DCN^a^	^13^C-MeOH	52,787±69	58% ^13^C^a^	nd
11) ^15^N^13^C, 0 .5% CN^b^	^13^C-MeOH	50,516±39	55% ^13^C	nd
12) ^15^N, 20% ^13^C	20% ^13^C-MeOH	49,626±56	14–16% ^13^C	nd
13) ^2^H^15^N, No sup.	^13^C-MeOH	52,287±81	36% ^13^C^a^	58%
14) ^2^H^15^N, 0.4% DCN^a^	^13^C,d_4_-MeOD	53,313±52	85% ^2^H/85% ^13^C^c^	93%

nd: not determined, insufficient sample

a DCN: ^2^H,^13^C,^15^N-ISOGRO^®^

b CN: 13C, ^15^N-ISOGRO^®,^ 85% deuteration and 100% ^15^N-incorporation assumed for the ^13^C-enrichment calculation
